# Outside in: Unraveling the Role of Neuroinflammation in the Progression of Parkinson's Disease

**DOI:** 10.3389/fneur.2018.00860

**Published:** 2018-10-15

**Authors:** Paulina Troncoso-Escudero, Alejandra Parra, Melissa Nassif, Rene L. Vidal

**Affiliations:** ^1^Faculty of Sciences, Center for Integrative Biology, Universidad Mayor, Santiago, Chile; ^2^Biomedical Neuroscience Institute, Faculty of Medicine, University of Chile, Santiago, Chile; ^3^Program of Cellular and Molecular Biology, Institute of Biomedical Sciences, University of Chile, Santiago, Chile; ^4^Geroscience Center for Brain Health and Metabolism (GERO), Santiago, Chile; ^5^Neurounion Biomedical Foundation, Santiago, Chile

**Keywords:** Parkinson's disease, neuroinflammation, gut microbiota, neurotrophic factor, neurodegenerative disease

## Abstract

Neuroinflammation is one of the most important processes involved in the pathogenesis of Parkinson's disease (PD). The current concept of neuroinflammation comprises an inflammation process, which occurs in the central nervous system due to molecules released from brain-resident and/or blood-derived immune cells. Furthermore, the evidence of the contribution of systemic delivered molecules to the disease pathogenesis, such as the gut microbiota composition, has been increasing during the last years. Under physiological conditions, microglia and astrocytes support the well-being and well-function of the brain through diverse functions, including neurotrophic factor secretion in both intact and injured brain. On the other hand, genes that cause PD are expressed in astrocytes and microglia, shifting their neuroprotective role to a pathogenic one, contributing to disease onset and progression. In addition, growth factors are a subset of molecules that promote cellular survival, differentiation and maturation, which are critical signaling factors promoting the communication between cells, including neurons and blood-derived immune cells. We summarize the potential targeting of astrocytes and microglia and the systemic contribution of the gut microbiota in neuroinflammation process archived in PD.

## Introduction

Parkinson's disease (PD) is the second most frequent neurodegenerative disease worldwide, affecting approximately 1% of adults whose age exceeds 50 years. PD is characterized by classical symptoms including bradykinesia, rigidity, tremor and later postural instability (1). Some non-motor symptoms such as depression, dementia, anxiety olfactory dysfunction and sleep disorders are also associated with PD and may precede motor symptoms by more than a decade, involving several neurotransmitter pathways beyond dopaminergic projections (2–5).

PD is caused by dysfunctions of the nigrostriatal pathway, which involve the loss of dopaminergic neurons in the Substancia Nigra pars compacta (SNpc) and the following loss of the dopamine circuit in the striatum (6). The onset of the cellular neuropathology of PD appears decades before the onset of the motor symptoms. Around 30% of the dopaminergic neurons are lost when the first symptoms of PD occur (5, 7). However, the cause of PD remains unknown. In less than 10% of PD cases, the disease is associated to genetic mutations (familiar Parkinson's), such as the mutation of the alpha-synuclein (*SCNA*) gene that encodes for the alpha-synuclein (α-syn) protein (8, 9). In the other 90% of the cases, the causes of the disease are unknown (idiopathic Parkinson's). Although mutations in the *SCNA* gene are not the most frequent mutations that cause familial PD, idiopathic cases of PD also show overexpression of the wild-type α-syn (10–12, 13). The misfolded protein α-syn is present in presynaptic cells as cytoplasmic inclusions named Lewy bodies, which are a biological hallmark of PD (14). Also, vast evidence shows a toxic effect of misfolded α-syn, particularly in dopaminergic neurons (15–18).

Despite the advance in our understanding about PD pathogenesis in the last decades, several details are still missing, hampering the rational development of therapies interfering with the processes underlying neuronal degeneration. Current therapeutic approaches provide symptomatic relief but fail to stop or slow down the course of the disease. In addition, the diagnosis of PD relies primarily on the clinical assessment of motor symptoms that become detectable only when a large part of the nigral dopaminergic neurons have already degenerated. Thus, unravel novel effective therapies that can slow down or reverse disease progression, specifically dopaminergic neurodegeneration, are urgently required.

In the ceaseless search for new therapies for PD, neurotrophic factors (NFs) have demonstrated to exert neuroprotection in animal models of PD, and are also under different phases of clinical trials as a treatment for PD patients (19–23). NFs are molecules produced mainly by neurons, which mediate synaptic plasticity, neuroprotection, neurorestoration and maintenance of neuronal functions. Moreover, after neural injury, some NFs facilitate tissue regeneration via their anti-inflammatory, anti-apoptotic, re-myelination and axon regeneration properties as well as by promoting adult stem cells to contribute to tissue repair (24). These molecules are also secreted by glial cells like microglia and astrocytes, which activate survival signaling pathways in neurons. Bidirectional communication between glial cells and neurons is critical to maintaining brain homeostasis. A loss in this communication occurs in the brain of PD patients, which cause the development of neuroinflammation observed in PD. In the following sections, we summarize the role of neuroinflammation in PD progression, as well as the implication of NFs from the central nervous system to this process.

## Inflammation in PD: cause or consequence of the disease?

The central nervous system (CNS) has long been considered a privileged immune tissue due to (a) the absence of dendritic cells, (b) the presence of an immunosuppressant microenvironment in the brain parenchyma under physiological conditions and (c) the presence of the blood-brain barrier (BBB) that separates the brain parenchyma and the peripheral immune system (25). Despite this, the CNS can initiate an immune response against insults such as pathogens or endogenous danger signals. This response is initiated by microglia, the resident tissue macrophages of the CNS, which can be activated by various stimuli (26). All the inflammatory reaction must be terminated to maintain the tissue structure and homeostasis, including the elimination of pathogens, dead cells or other cellular debris, and tissue restoration. If the insult persists or the mechanisms involved in the termination of the inflammation are inadequate, chronic inflammation can arise (27). Furthermore, inflammation can also occur in response to secreted molecules from neurons under degeneration, a condition called neuroinflammation, a crucial player in neurodegenerative diseases (28). If neuroinflammation is a cause or consequence of neurodegenerative diseases, it remains unknown.

### Neuroinflammation: hallmarks in parkinson's disease

In PD, as well as in other neurodegenerative diseases, there is dysfunction and loss of specific neurons in a specific region of the brain. Several mechanisms have been described as triggers of neurodegeneration that are common among neurodegenerative diseases, such as protein aggregation due to protein misfolding or no degradation, formation of reactive oxygen species (ROS) and reactive nitrogen species, which causes oxidative stress, mitochondrial dysfunction, endoplasmic reticulum stress, dysfunction of neurotrophic factor, chronic neuroinflammation, among others (29). Inflammation is a highly regulated self-defensive mechanism against pathogenic stimuli or injury, generated by an activated immune system that seeks to protect the host organism and get rid of the pathogenic stimuli to promote a healing process (30). The immune system can be classified as innate or adaptive. The innate immune system is the first line of defense against insults, creating a rapid but short-term response. Mononuclear phagocytes (dendritic cells, macrophages, microglia, and monocytes), natural killer cells and neutrophils are responsible for triggering this response. On the other hand, the adaptive immune system generates a pathogen-specific, non-rapid and long-lasting response, in which T- and B-lymphocytes are responsible (30).

Under physiological conditions, inflammatory molecules are not expressed, given that the expression of their genes is suppressed. However, under a stress condition such an infection or necrosis signals, non-self-molecules are recognized by pattern recognition receptors such as Toll-like receptors (TLRs) and nucleotide oligomerization domain receptors (NLRs) (31). Stranger molecules that are specific to bacteria or virus are collectively known as pathogen-associated molecular patterns (PAMPs), whereas endogenous molecules that came from the host cells are known as damage-associated molecular patterns (DAMPs), and include chromatin, adenosine, ATP, heat-shock proteins, β-amyloid, tau, α-syn, among many others (32, 33). Upon the presence of PAMPs and/or DAMPs, TLRs and NLRs are activated in microglia and astrocytes, which secrete NFs that promote tissue repair and regrowth (32).

Studies in experimental animal models of PD have shown that neuroinflammation plays a key role in disease progression (34). It has been demonstrated the interplay between neuroinflammation and other proposed pathogenic mechanisms of PD, such as mitochondrial dysfunction and oxidative stress (35), the participation of protein products of parkinsonian genes, such as α-syn, Parkin and DJ-1 in innate immune responses (36–39). An immunoregulatory role of dopamine has also been described during neuroinflammation (40). Inflammatory responses may also contribute to the intrinsic vulnerability of nigral dopaminergic neurons, explained by several factors such as dopaminergic metabolism, high iron content, differential transcriptional profile, different calcium channel expression and a low antioxidant defense system (6).

**Figure 1:**
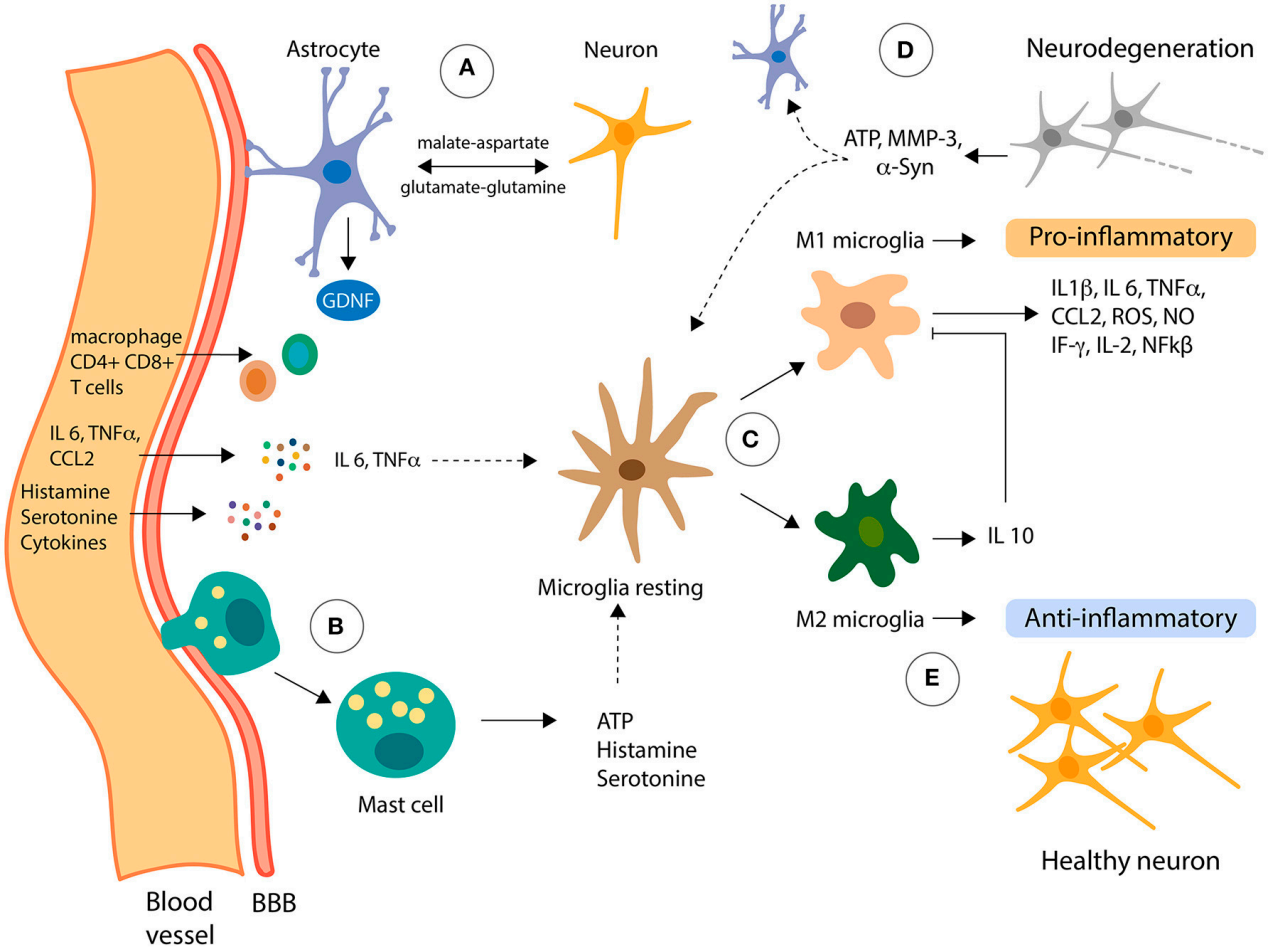
Role of non-neuronal cells in neuroinflammation. Pro-inflammatory molecules can reach the Central Nervous System (CNS) from the periphery going across the blood-brain barrier (BBB). **(A)** Astrocytes, the most abundant cells in the CNS, are functionally connected with the BBB, receiving signals from the periphery and from inside the CNS. Also, astrocytes metabolically support neurons via the shuttle systems malate-aspartate and glutamate-glutamine. **(B)** Mast cells can infiltrate the CNS, inducing changes in the microglia by the delivery of proinflammatory effectors, including ATP, which stimulates transcription of proinflammatory cytokines through PKC. **(C)** Resting microglia can be activated in two classical phenotypes, M1 and M2, depending on the effector signals from its microenvironment. **(D)** In presence of LPS and IFNγ, microglia cells polarize to M1 phenotype and secrete the proinflammatory cytokines which contribute to the dysfunction of dopaminergic neurons (neurodegeneration). Moreover, neuron failed can release α-Syn, ATP, MMP-3, among other molecules, in a cross-talk signaling with astrocytes and microglia, increasing the toxic-loop of neuroinflammation. **(E)** Contrary, IL 4 and IL 13 induce activation of microglia to M2 phenotype that downregulates M1 functions by release of IL 10 cytokines contributing to anti-inflammatory of CNS.

Since many studies have demonstrated the complex neuroimmune interactions occurring both at homeostatic and pathological conditions in the CNS, the notion of the CNS as a tissue immune privilege has been refined (25). For example, in PD the integrity of the BBB is compromised, and the components of the innate immune system are activated, allowing the recruitment and activation of the adaptive arm of the immune system [(25); Figure 1]. While the role of the immune system is not clear and has not been extensively studied in the etiology of PD, it is well known that the immune system is critical for the progression of the disease (41–43). The initial activation of the innate immune system may have protective roles, but when these innate defense mechanisms become dysregulated and maladaptive, it leads to disease progression.

### Role of microglia in neuroinflammation

Microglia are the immune cells of the CNS, constituting 5-10% of total brain cells and the 20% of the glial cell population of the brain (26). Microglia are in a quiescent state in the absence of any stimulus, which is achieved by the immunosuppressant microenvironment present in the CNS, where immunoregulatory molecules are expressed and/or released by healthy neurons (44). Among these immunoregulatory molecules are CX3CL1, CD200, CD22, CD47, CD95 and neuronal cell adhesion molecule (NCAM), and the receptor for these molecules are almost exclusively expressed in microglia, evidencing the important role of neuron-microglia interactions in the regulation of neuroinflammation (45). This communication between neurons and glia are of special importance because microglia have the potential to damage brain tissue, which has limited capacity for regeneration and repair. Microglia are the responsible cells for mediating the innate immune response in the brain through antigen-presenting and effector functions such as phagocytosis (26). Besides its immunological functions, microglia play other roles that are beneficial for neurons, such as NFs release, removal of toxic substances, neuronal repair, synaptic remodeling and synaptic pruning (26). Microglia localize in specific structures in the human brain, including the medulla oblongata, pons, basal ganglia, and SNpc (46). In addition to microglia, which are in the brain parenchyma, the CNS contains other types of mononuclear phagocytes, which are meningeal macrophages, choroid plexus macrophages, epipexus cells and perivascular macrophages (26).

Microglial activation can be triggered in response to a variety of environmental challenges, a process that involves morphological changes and upregulation of a spectrum of intracellular molecules and surface antigens. Microglia can be activated by bacterial and viral molecules, as well as with disease-related proteins (amyloid β and α-syn) and soluble molecules released by dying neurons (47). When microglia are activated, transformation and proliferative events take place to form reactive microglia (48), which are distinguished classically by two distinct phenotypes: M1 phenotype (pro-inflammatory) and the M2 phenotype (anti-inflammatory) (Figure 1). During transformation, the resting ramified phenotype of microglia changes into an intermediate hyper-ramified morphology, which consists of a large soma and an amoeboid morphology to initiate phagocytosis (49). Together with this changes, microglia upregulate cell surface markers of inflammation, including MHC class I and II, and cytokine and chemokine receptors (48). Acute or chronic activation of microglia can occur, depending on the type and duration of the external stimuli or activated factor (50). Short-term activation of microglia is generally believed to be neuroprotective, while chronic activation has been implicated as a potential mechanism in neurodegenerative diseases (51). The mechanism that underlies the change from a neuroprotective to an autoaggressive effector microglia, which causes neurodegeneration, has long been elusive, but recent findings are shedding light on the mechanisms involved in this change (52). In their work, Du et al. describe that the deficiency of Kir6.1-containing ATP-sensitive potassium (Kir6.1/K-ATP) channel favors the M1 phenotype which exacerbates the inflammatory response and dopaminergic neuronal loss in a MPTP model through the activation of p38 MAPK–NF-kβ pathway and the increasing the ratio of M1/M2 markers in SNpc (52).

Several studies have shown that dying neurons release soluble mediators, such as α-syn, matrix metalloproteinase-3 (MMP-3), neuromelanin, ATP and m-calpain, which cause the secretion of toxic mediators by microglia that are lethal to neighboring cells and stressed neurons (53). Pro-inflammatory mediators released by activated astrocytes act on their cognate receptors expressed in microglia and further increase the microglial activation by rendering them to an overactivated state (Figure 1). Moreover, misfolded proteins induce the activation of microglia toward an M1 phenotype in *in vitro* and *in vivo* models of PD (54–56). For example, chronic administration of MPTP leads to a reduction of CD206, a molecular marker of M2 microglia, suggesting downregulation of this phenotype activation in this model of PD (57). Cell culture experiments have demonstrated that dopaminergic neurons incubated with conditioned medium (CM) from M1 microglia increase the death of these neurons, whereas a mixture of CM from both M1 and M2 microglia reverses the neurotoxicity elicited by the M1-CM (58).

### Role of astrocytes in neuroinflammation

Astrocytes are the most abundant glial cells in the CNS and are five times the number of neurons (59). Astrocytes have numerous extensions that connect directly with neurons and blood vessels of the BBB to form a functional network via gap junctions, which is called the neurovascular unit (NVU) (60). Given this phenomenon, astrocytes participate in the maintenance and permeability of the BBB and are key regulators of neuronal activity and cerebral blood flow (Figure 1). Metabotropic glutamate receptors in the membrane of astrocytes release arachidonic acid metabolites, which causes an increase of calcium levels as a result of activating the inositol triphosphate (IP3) pathway at the astrocyte end feet. If this activation occurs near a blood vessel, it results in the dilation of blood vessels (61). Moreover, astrocytes give biochemical and nutritional support to neurons, extracellular ion balance, and repair of scarring of the brain and spinal cord tissues (60). Astrocytes also produce and secrete NFs, including the glial-derived neurotrophic factor (GDNF) (Figure 1), which is especially important for the development and survival of dopaminergic neurons (62). Also, via specific shuttle systems, such as the malate-aspartate and glutamate-glutamine shuttle systems, astrocytes transport nutrients and metabolites to neurons (63, 64) (Figure 1).

*In vitro* and *in vivo* studies show the vital role that astrocytes play in the neuroinflammatory processes in PD. Astrocytes, like microglia, respond to inflammatory stimulations such as IL-1β, LPS, and TNF-α, producing more proinflammatory cytokines (65, 66). Reactive astrogliosis has been reported in different PD animal models, and importantly, in the affected brain regions of PD patients, indicating a possible involvement of astrocytes in the immune response in PD. Treatment of astrocytes primary culture with α-Syn increase the expression levels of IL-6 and TNF-α (67) and overexpression of mutant α-Syn in astrocytes causes astrogliosis, microglial activation and degeneration of dopaminergic neurons and motor neurons in mice (68).

As a consequence of brain diseases such as infection and neurodegeneration, and brain injuries like trauma and ischemia, astrocytes become reactive, a process known as reactive astrogliosis. Reactive astrocytes experience changes in gene expression (69) and in morphology (70), which leads to the formation of a glial scar in the site of injury. For a long time, it has been debated if reactive astrogliosis is beneficial or detrimental for the recovery of the injured CNS. Several studies have demonstrated that reactive astrocytes can play both roles (71–74), which raises the question of whether there are different populations of reactive astrocytes activated by different stimulus, and which can in turn, have different functions. This question was addressed by Zamanian group who elegantly and meticulously demonstrated that reactive astrocytes gene expression differs depending on the brain injury model used: focal ischemic stroke produced by transient occlusion of the cerebral middle artery (MCAO) or neuroinflammation induced by systemic LPS injection (75). The authors obtained pure reactive astrocytes from both models, and identified a total of 263 reactive glial genes, 150 of which were preferentially expressed by MCAO reactive astrocytes, 57 were preferentially expressed by LPS reactive astrocytes, and 56 genes were shared, including GFAP and vimentin, classical markers of reactive astrocytes (75). Importantly, the authors identify a new set of genes induced under both brain injury studies, which can now be used as new markers for reactive astrogliosis. Finally, the authors hypothesized, based on their results and the findings by Sofroniew group (69), that MCAO reactive astrocytes are protective, given the expression of high levels of neurotrophic factors and cytokines, which may help repair and rebuild damaged synapses (76). On the contrary, the authors postulated that LPS reactive astrocytes may be harmful, due to the upregulation of genes for the classical complement cascade, which is thought to cause synapse loss and neuronal loss in neurodegenerative diseases (77). Therefore, reactive astrocytes induced by neuroinflammation are termed A1 reactive astrocytes, and those induced by ischemia are termed A2 reactive astrocytes, in analogy to the M1/M2 macroglia nomenclature.

Recent findings have uncovered how A1 reactive astrocytes are activated under LPS stimulation. Starting with the premise that A1 reactive astrocytes are induced by LPS and that LPS is an activator of microglia through TLR4. Liddelow group demonstrated that LPS-activated microglia secreted IL-1a, TNF and complement component 1, subcomponent q (C1q) (78) which changes astrocytes toward an A1 phenotype nearly identical to the phenotype found by LPS induction *in vivo* (75). Furthermore, astrocytes activation is completely dependent on the presence of microglia, because LPS treatment of animals lacking microglia, or pure astrocytes *in vitro* cultures, fails to achieve the activation of astrocytes. Also, the authors showed that LPS treatment of a triple knockout mouse for IL-1a, TNF and C1q causes no A1 reactive astrocytes, further confirming that these molecules are necessary and sufficient to activate astrocytes toward an A1 phenotype. Another important finding is that A1 reactive astrocytes show loss of normal astrocytes function and a gain of toxic functions. A1 reactive astrocytes lose the ability to form functional synapses *in vitro* and loose phagocytosis activity both *in vitro* and *in vivo*. On the other hand, neurons cultured with A1 astrocytes show increased percentage of cell death, which account for a loss of the protective role of astrocytes on neuronal survival. The finding that IL-1a and TNF secreted by activated microglia is able to activate astrocytes toward an A1 phenotype, which causes neuronal loss and possibly contributes to neurodegeneration seen in different brain diseases, opens the possibility for the study using antibodies against both molecules, which are already approved by the FDA for the treatment of others maladies.

Several studies have demonstrated that LPS injection is sufficient to cause dopaminergic neurons degeneration that simulates PD (79). Human dopaminergic neurons cultured together with A1 astrocytes show a 25% increase in cell death, which is attributed to the activation of apoptosis in these neurons (78). Furthermore, analysis of human post-mortem tissues shows an important amount of A1 reactive astrocytes in the brain areas affected in different neurodegenerative diseases, such Alzheimer's disease, Parkinson's disease, Huntington's disease, amyotrophic lateral sclerosis and multiple sclerosis. The presence of these cells, together with activated microglia, could be accounting for the selective neurodegeneration seen in these diseases, which is accompanied with neuroinflammation, which furthers enhances neuronal loss.

Increasing evidence suggests that disruption of astrocyte biology is involved in dopaminergic neuron degeneration in PD. As mentioned before, monogenic mutations in 17 genes have been identified in the development of the disease, and many of these genes are expressed in astrocytes at levels comparable to, and even higher than, in neurons (80). Recently, proteins encoded by eight of these genes have been shown to have a role in astrocyte biology [reviewed in (81)].

Overall, it is clear that microglia and astrocytes play important roles in the maintenance of the CNS homeostasis, and these neuroprotective roles are lost under brain injury. Also, it is clear that both glial cells are constantly interacting, where activated microglia can activate astrocytes toward a neurotoxic phenotype (78). For many years, therapies aimed to slow down or stop CNS diseases have neurons as the principal objective. With these new findings, it becomes increasingly interesting to target microglia and astrocytes, given that the prevention of glia activation has a positive outcome in neuronal survival. Recently, it has been demonstrated that the GLP1R agonist NLY01 protects against dopaminergic neurons loss in two models of PD (82). Interestingly, this protection was due to the prevention of astrocytes A1 activation by activated microglia, not by a direct effect of NLY01 on neurons. Targeting glial cells may be the next step in the development of therapies for the treatment of different CNS maladies.

### *In vivo* evidence of neuroinflammation in PD

The most widely used preclinical model of PD is based in the administration of 6-hydroxydopamine (6-OHDA), a selective catecholaminergic neurotoxin that upon injection into the striatum, causes retrograde degeneration of the nigrostriatal dopaminergic circuit (83). Because 6-OHDA cannot cross the BBB, it has to be injected into the brain by stereotaxic surgery. The neurotoxic effect of 6-OHDA is due to the oxidative stress triggered by ROS production (83). Cellular and molecular evidence of inflammation is observed in the 6-OHDA-induced animal model of PD. Intranigral 6-OHDA injection in mice generates acute astrogliosis and microgliosis in the nigrostriatal system, which is accompanied by degeneration of nigral dopaminergic cell bodies (84). Reactive microglia precede astrogliosis, demonstrated using GFAP immunohistochemistry, and these active microglia upregulates the expression of the gene coding for TNF-α, a pro-inflammatory molecule known to drive the progression of neurodegeneration (85). A recent study demonstrated that unilateral injection of 6-OHDA in the striatum of mice induces an increase in the levels of the pro-inflammatory cytokines TNF-α, IF-γ, IL-1β, IL-2, IL-6, and NF-kβ, in parallel with a decrease in the levels of the anti-inflammatory cytokine IL-10 in the striatum of these mice (86). Importantly, this was reversed when mice were treated with Chrysin, a natural flavonoid known to have neuroprotective effects (87, 88).

Another potent neurotoxin used to mimic PD in a wide range of organisms including non-human primates, guinea pigs, mice, dogs and cats is 1-methyl-4-phenyl-1,2,3,6-tetrahydropyridine (MPTP) (83). Because MPTP is a lipophilic molecule, it rapidly crosses the BBB and is converted by astrocytes into the toxic metabolite MPP^+^ (89). MPP^+^ is released by striatal and nigral astrocytes and is taken up by dopaminergic neurons through the dopamine receptor. Inside neurons, MPP^+^ induces neurotoxicity by inhibiting the mitochondrial electron transport chain complex I, resulting in ATP depletion and increased oxidative stress (90). Inflammation markers have also been evaluated in the MPTP model. Mice treated with MPTP have a significant increase in the pro-inflammatory cytokines IL-1, TNF-α, and IL-6 mRNA levels both in the SNpc and striatum (91). Additionally, both mRNA and protein levels of the receptors for these three cytokines were increased in the SNpc of MPTP-treated mice, although this increase was not observed in the striatum of these mice (91). Rai et al. have demonstrated an increase of the inflammation-related molecules GFAP, iNOS, ICAM, and TNF-α in the SNpc of mice treated with MPTP (92). This increase was reverted when MPTP-treated mice were administered with *Macuna pruriens* (Mp) seed, which have been previously demonstrated to be neuroprotective in a mouse model of MPTP (93). In MPTP-treated mice, Mp also inhibited the activation of NF-kβ and promoted the activation of pAkt1, preventing the apoptosis of dopaminergic neurons (92). A decrease in the mRNA levels of the pro-inflammatory cytokines IL-1β and TNF-α was also seen in mice treated with MPTP and naringenin, another natural product (94). The use of plant-derived natural products to treat PD have been extensively studied, but its effects in neuroinflammation have to be further investigated. Blocking microglial activation by minocycline also protects the nigrostriatal dopaminergic pathway against MPTP model, suggesting that microglial activation plays a crucial role in the pathogenesis of PD (95). The treatment with paeonol decreased MPTP/p-induced oxidative stress and neuroinflammation through the increase of the brain-derived neurotrophic factor (BDNF), one of the most critical NFs in the physiology of CNS (96).

## The anti-inflammatory contribution of neurotrophic factors in preclinical models of PD

As described above, an inflammatory response in the CNS mediated by activated microglia and astrocytes contributes to neuronal degeneration in PD. For this reason, the incessant search for therapeutic alternatives with neuroprotective and anti-inflammatory effects is a relevant consideration in research. Several NFs have been considered as an alternative for the treatment of neurodegenerative pathologies such as PD. However, only subsets of NFs have shown to be neuroprotective and neurorestorative in pre-clinical animal models of PD, highlighting among them the glial cell line-derived neurotrophic factor (GDNF), cerebral dopamine neurotrophic factor (CDNF) and vascular endothelial growth factor (VEGF).

GDNF belongs to the TGF-β superfamily of NFs (97), which was first purified from a rat glioma cell line (B49) medium and identified to promote the survival of embryonic dopaminergic neurons in culture and increase dopamine uptake (62). GDNF is considered the most potent neuroprotective agent tested in cellular and animal models of PD (62, 98). The neuroprotective and neurorestorative effect of GDNF has been shown in numerous neurotoxic PD models including mouse, rat (99–101) and no-human primates (102). In a recent study, Chen et al. demonstrated that GDNF derived from macrophages diminished the loss of dopaminergic neurons and improved motor symptoms in a mouse model of PD (103). In this study, bone marrow hematopoietic stem cells were transduced with a lentiviral vector expressing macrophage promoter-driven GDNF and transplanted into the MitoPark mice (103, 104). These genetically modified macrophages were able to infiltrate the midbrain of MitoPark mice, but not control littermates. This was accompanied with GDNF secretion, an improvement in motor and non-motor symptoms and a reduction of dopaminergic neurons loss in the SNpc and its axonal terminals in the striatum (103). The mechanisms that are involved in the neuroprotective effect of GDNF are still unknown. *In vitro* and *in vivo* assays suggest that GDNF can protect from neurodegeneration thought the inhibition of neuroinflammation. Using an inflammatory model of PD based on LPS treatment, it has been demonstrated that GDNF delivery by mesenchymal stem cells provides localized neuroprotection of dopaminergic neurons (105). Additionally, in a neurotoxic model of PD, the intracerebral administration of GDNF by microspheres reduced the TNF-α levels, an important pro-inflammatory cytokine involved in neuronal death (98, 106). Using an *in vitro* assay, it was demonstrated that astrocyte-derived GDNF is an inhibitor of the activation of microglia. In this experiment, midbrain microglia cultures were incubated with astrocytes conditioned media that reduced microglial activation, however, when the medium was neutralized with GDNF antibody the effect was abrogated (107). However, in genetic PD models generated by overexpression of mutant or wild-type α-syn into the midbrain, it has been reported that GDNF fails to exert robust neuroprotection (21, 108). According to the later, overexpression of α-syn would cause an alteration in GDNF signaling and a decrease in its neurotrophic effects in dopaminergic neurons (108). Nevertheless, it has been recently demonstrated that α-syn accumulation does not block the expression of GDNF in patients and preclinical models of PD (109). More recently, clinical application of GDNF (clinical trial phase 2) has failed to demonstrate a significant positive effect (110). New approaches for GDNF administration are being tested in animals models (103). For example, the intrastriatal infusion of a variant of GDNF, which was designed to promote a better tissue distribution and to enhance its chemical stability, increased the dopamine turnover and protected midbrain dopaminergic neurons function in 6-OHDA-lesioned rats (110). In mice model of PD, intravenous-injected GDNF-transfected macrophages can cross the blood-brain barrier, reduce microglial activation and the loss of dopaminergic neurons in the SNpc, to improve the motor dysfunction observed in 6-OHDA-lesioned mice (111).

CDNF is an unconventional neurotrophic factor that presents a robust effect in reducing dopaminergic neurons loss (112, 113), along with the ability to promote neurorestoration in a neurotoxic model of PD (114). Recently, it has been reported the neurorestorative effect of CDNF by its single administration or as co-treatment with subthalamic nucleus deep brain stimulation (STN-DBS), which might be explained by the interaction of electric stimulation and NFs (115). CDNF can regulate ER stress and exhibit anti-inflammatory properties that promote neuronal survival (24, 116). In *in vitro* assay, the overexpression of CDNF reduces the cytokine secretion by astrocytes under ER-stress (117). Moreover, overexpression of human CDNF into the rat SNpc reduces de levels of glial markers and IL-6 in pharmacological model of PD (118). In LPS-treated microglial cultures it has been showed that CDNF treatment has anti-inflammatory effects, attenuating the production of pro-inflammatory cytokines and cytotoxicity by inhibition of JNK signaling (119). Additionally, *in vitro* assays revealed that CDNF protects against toxicity induced by α-syn oligomers in primary cultures of mesencephalic neurons (120).

The members of the VEGF family are key regulators of vascular biology, modulating angiogenesis, vasculogenesis, and maintaining vasculature during embryogenesis and in adults (121, 122). However, the neuroprotective role of this growth factor family for the treatment of neurodegenerative diseases has also been studied (122). VEGF-A is the most studied of the VEGF family, highlighting its angiogenic role (123). Additionally, it has been reported it neuroprotective effect both *in vitro* and *in vivo* in PD models (124–126). However, an increase in the levels of VEGF-A contributes to the development of L-DOPA-induced dyskinesia (LID), which has been associated with its angiogenic effect (127, 128). On the other hand, VEGF-B has emerged as an alternative for the treatment of neurodegenerative diseases, given its anti-apoptotic effects in different cell types, by suppressing the expression of genes related to apoptosis and its angiogenic effect (129, 130). *In vitro* assays have shown that exogenous administration of VEGF-B reduces neuronal loss in a PD model generated by the addition of Rotenone (131), a toxin used as a pesticide that reproduces the pathological characteristics of PD in cellular and animal models (132, 133). VEGF-B has also shown a neuroprotective effect in an animal model of PD, which is accompanied by an improvement in motor symptoms, but with no changes regarding dopaminergic neuronal loss (101, 134). However, its use in combination with other neurotrophic factors such as GDNF in nanoparticles, has shown a synergistic effect, favoring neuroprotection and neurorestoration processes (135). Although the use of VEGF has shown clear neuroprotective effects in PD pharmacological models, clinical trials using this therapeutic target have not yet been carried out.

### Neurotrophic factors as therapeutic targets for PD

Currently, the success of the NFs application in clinical trials has been modest. This could be explained considering that PD preclinical models present a partial lesion (early stage PD) unlike the patients condition, which receive this alternative treatment after the onset of motor symptoms (late-stage PD), with a 80% decrease in dopamine content in the striatum, 50–80% loss of striatal dopaminergic innervations and a 30% loss of dopaminergic neuron in SNpc (136–138). Another challenge in the clinical use of NFs for the treatment of neurodegenerative diseases is their inability cross the BBB. Direct needle or catheter delivery has a limited clinical use. Non-invasive drug delivery for early-stage patients throughout the diseased regions may be critical to improve patient response.

## Extracerebral origin of parkinson's disease: does alpha-synuclein reach the brain from peripheral organs?

### Role of the peripheral system in the pathogenesis of parkinson's disease

As mentioned before, the CNS was considered for many years to be immune-privileged, being excluded from the effects of immune-molecules released by inflammatory cells from systemic reactions. However, the process of “neuroinflammation” is a consequence of the complex signaling between systemic and CNS cells. The players are the inflammatory substances that arrive to the CNS from the periphery, the infiltrated mast cells or T-lymphocytes after the delivery of chemoattraction substances or rupture of the BBB and the sustained activation of glial cells in the CNS (microglia, astrocytes and even oligodendrocytes) [reviewed in (139, 140)]. Astrocytes, the cells that are functionally connected with the BBB by surrounding the endothelial cells in the brain, can be activated directly by molecules from the circulation, and secrete pro-inflammatory molecules and NFs (Figure 1). In PD, for instance, the loss of dopaminergic neurons was shown to be accompanied by activated microglia and T-cells infiltration (141). Indeed, it was recently shown a pathway in which activated microglia release cytokines that stimulated MHC-I expression in dopaminergic neurons, which finally are attacked by T-CD8^+^ cells (142). Another player in the connection between the peripheral system and the CNS is the gut microbiota, with surprising incidence in the development of neurodegenerative diseases. In the next section, we will review the recent findings about the gut microbiome alteration on PD and its implication en the progression of this disease.

### Impact of the enteric nervous system in PD

Although studies of neurodegenerative diseases have historically been performed in brain tissue, the influence of peripheral organs emerges as an important niche to study and understand the origin and/or progression of diseases affecting the CNS, giving the direct contact between gut neurons and the CNS (143). Recently, there has been an increase in the number of studies describing close bidirectional communication between the gut and the brain in neuropsychiatric disorders such as anxiety, depression, autism, among others (144–146). Additionally, gastrointestinal physiology is influenced by signals generated both locally in the intestine and from the brain. Neurotransmitters, immune signaling, hormones, growth factors (GFs) and neuropeptides produced in the intestine can, in turn, affect the brain (147, 148).

From clinical studies, it has been described that PD patients present intestinal inflammation (149) and gastrointestinal anomalies, such as constipation, which often precedes for many years the motor deficits characteristic of this disease (150, 151). The Braak hypothesis suggests that the aberrant accumulation of the α-syn protein starts in the intestine and spreads through the vagus nerve to the brain like a prion disease (151). This idea is supported by surprising physiopathological evidence that describes the presence of protein inclusions of α-syn in the Enteric Nervous System (ENS) and in the glossopharyngeal and vagal nerves in early stages of PD (152). Additionally, vagotomized individuals have a lower risk of developing PD (153).

The concept regards to the extracerebral origin of PD is becoming increasingly relevant. For example, the injection of α-syn fibrils into the intestinal tissue of healthy rodents is sufficient to induce pathology in the vagus nerve and brainstem (154). Due to the immediate proximity of the ENS to feces, the gut microbiota and the metabolic products of the microbiota, are presented as potential candidates that could initiate a process that eventually results in the formation of α-syn protein aggregates in the ENS and that this spreads to the brain.

### Alterations of the intestinal microbiota in parkinson's disease

Microorganisms permanently colonize the human body in virtually all environmentally exposed surfaces, where the most significant percentage of these reside within the gastrointestinal tract (155). Intestinal bacteria control the differentiation and function of immune cells in the intestine and the brain (156–158). The impact of gut microbiota on neurological development and neurodegenerative diseases emerges as an innovative alternative to understand the molecular processes that govern these complex biological processes (146).

Similarly, perturbations of the bidirectional network known as the “intestinal microbiota-brain axis” can affect brain physiology (159) and have been linked to numerous diseases (160). Alterations in the gut microbiota can affect both brain neurochemistry (altered levels of neurotransmitters, their receptors and various neurotrophic factors), as well as behavior (161–164). Recently, evidence has described the role of gut microbiota in the regulation of the expression levels of synaptic components, such as the 5-hydroxytryptamine (5-HT1A, serotonin) receptor, BDNF and the subunit 2 of the NMDA receptor (NR2A) (161, 162, 165). In addition, it can alter the enteric and circulating production of serotonin in mice (166), which in turn generates anxiety, hyperactivity and cognitive alterations (147, 162, 167, 168). These alterations of the gut microbiota, known as dysbiosis, have been observed in patients diagnosed with various neurological diseases (145).

**Figure 2:**
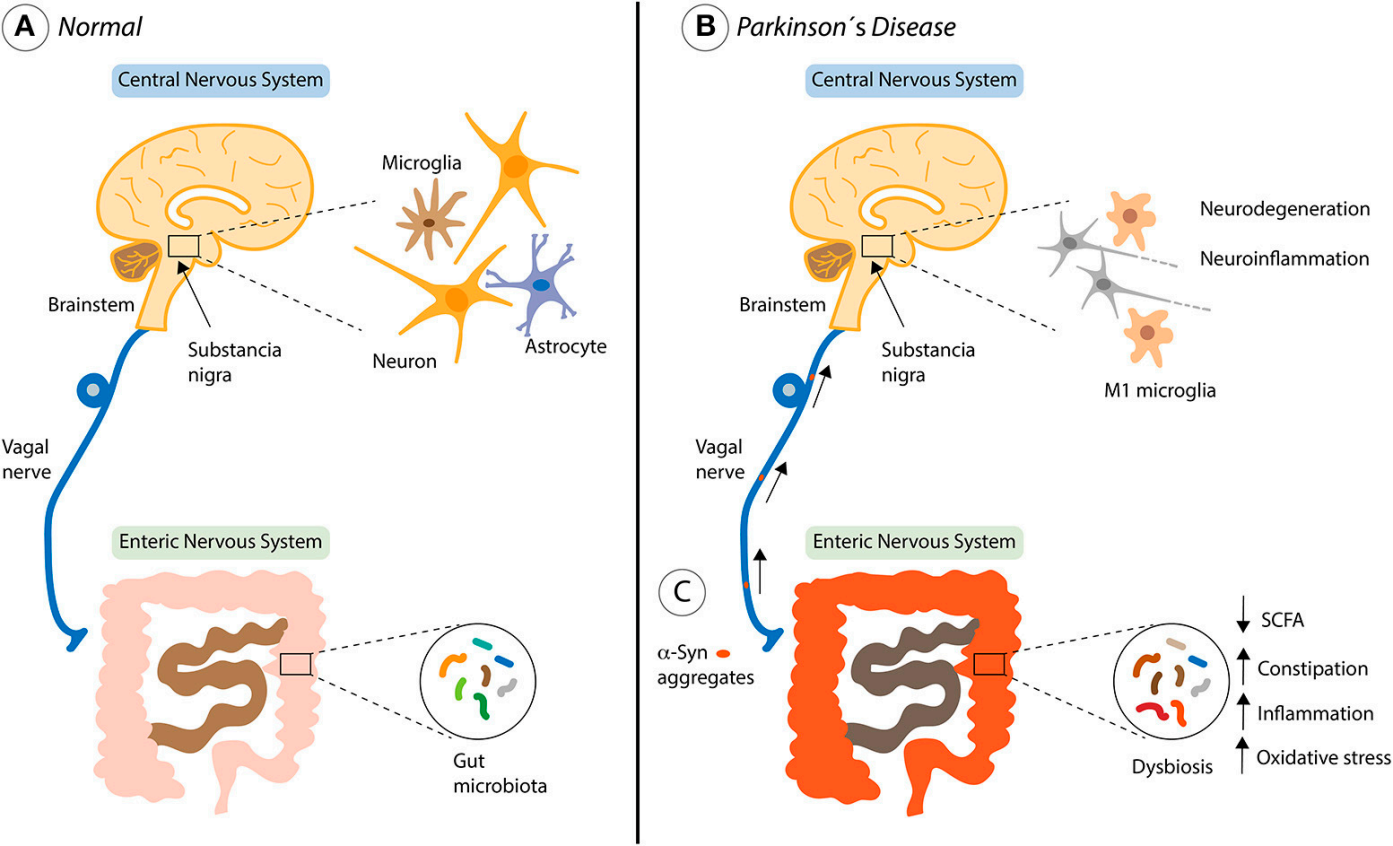
Contribution of the gut microbiota in Parkinson's disease progression. **(A)** The healthy bi-directional communication between the brain and the gut, highlighting the involvement of the vagus nerve. **(B)** The brain-gut axis and non-motor symptoms of Parkinson's disease (PD) including both central and gastrointestinal dysfunction. **(C)** Environmental factors such as the gut microbiota, might begin a pathological process within enteric nerve cell plexus, causing mucosal inflammation and oxidative stress, thereby initiating alpha-synuclein (α-syn) accumulation. The vagal nerve might provide a footpath for the spread of α-Syn from the Enteric Nervous System to the brain through the brainstem, Substancia nigra, basal forebrain and finally the cortical areas where is activated the neurodegeneration and neuroinflammation process described in PD.

In PD, it has been determined clear differences in the gut microbiome from PD patients and healthy people (169–171). Recent studies have described that alterations in the intestinal microbiota promote the pathology of α-syn, neuroinflammation and the motor symptoms of PD in a mouse model of this disease [Figure 2; (172)]. In this work, the authors performed fecal transplantation from PD patients to healthy mice, which generates a significant deterioration in the motor function of these animals (172). Surprisingly, they also identified specific metabolites of the microbiota present in the feces of patients that are sufficient to promote the PD symptoms. The gut microbiota is exclusively responsible for several metabolic functions, including the production of short chain fatty acids and vitamins (SCFAs), amino acid synthesis (AAs), biotransformation of bile acids, hydrolysis and fermentation of non-digestible substrates (173). In addition, the beneficial functions of gut microbiota include (i) homeostasis and development of immune system cells, (ii) homeostasis of epithelial cells, (iii) enteric nerves regulation and (iv) angiogenesis, food digestion and fat metabolism induction (160, 174). Interesting, mouse models of gut injury have shown that gut microbiota can penetrate injured areas and induce macrophages to migrate to the damaged sites, triggering the expression of specific GFs to recover tissue homeostasis (175).

The molecules that are produced by the microbiota can cross the epithelial barriers to cause systemic effects at distant sites of the organism. Moreover, the fermentation of dietary fiber by gut microbiota produce SCFAs, such as acetate, propionate and butyrate, which are absorbed by epithelial cells and used as an energy source (176). For instance, an association has been shown between the abundance of specific gut bacteria and PD development (177), where patients with PD have a decrease in the number of intestinal bacteria that are capable of producing SCFA. The SCFA can modulate the activity of the ENS and therefore increase gastrointestinal motility (178). Therefore, altered concentrations of SCFA could contribute to decreased gastrointestinal motility in patients with PD (171). The presence of metabolic biomarkers in the blood is especially useful for the diagnosis of diseases because they can reveal the physiological state of both the host and its microbiota (179, 180). Such biomarkers may correspond to the final products of the metabolism of microorganisms, providing mechanistic explanations for the association between changes in the microbiota and the development of the disease (171).

The appearance of gastrointestinal symptoms, the dysbiosis present in patients with PD, and the studies that show that the microbiota can affect brain functions, bring us to the conclusion that intestinal bacteria can regulate the progression of motor deficits and the pathophysiology observed in patients with PD.

### The olfactory bulb as a possible initial site of α-syn spreading

Although the trigger of PD pathogenesis is unclear, several hypotheses were outlined in the last years. One of them proposes that the beginning of the neurodegeneration in PD occurs in the olfactory bulb, in a called olfactory vector hypothesis (150, 181–183). Around 90% of the PD patients present a loss of the sense of smell in early stages of PD (184, 185), and this olfactory dysfunction is one of the first symptoms during disease progression, years before motor symptoms appear. The olfactory sensory neurons are bipolar neurons, in which dendrites are exposed to the exterior environment, and the axons project directly to the brain. It means that our olfactory mucosa is exposed for decades to the air components, and might be the via of entrance to the CNS of environmental contaminants, such as xenobiotics, viruses and metals. As an example of environmental contaminants is paraquat, a herbicide known to cause parkinsonism. In a recent study, a group of researchers showed for the first time significant structural differences between the olfactory bulb from PD patients and age-matched controls. The total volume occupied by the functional units of the olfactory bulb (glomeruli) in PD is around half that in controls (186). Remarkable, the researchers establish an indirect relationship between the volume of the olfactory bulb and the phosphorylation of α-syn: smaller the olfactory bulb, increased phospho-α-syn was found (186). Since the modification of α-syn found in the olfactory bulb neurons might predict the brain α-syn pathology in the CNS (187), and the olfactory dysfunction is presented years before the first motor symptoms of PD, this study supports the olfactory vector hypothesis, including the modifications of α-syn and its prion-like spread to the CNS (183).

The olfactory bulb could be the start or the intermediate point before arriving at the brain of other responses as well, such as the inflammatory response. For instance, a study conducted in rats showed that intravenously delivered LPS provoked a robust inflammatory response in the olfactory bulb, with the presence of peripherical immune cells and increased levels of pro-inflammatory cytokines, such as TNF-α, IL-1β, IL-6, and IL-10 (188). However, if it is enough to follow to the Snigra reaction, is unknow.

## Concluding remarks

Researchers continuously revisit the role of inflammation in the progression of neurodegenerative diseases. It was already known that during aging there is a decline in physiologic protective processes, vital in maintaining the body homeostasis. However, some pathways are persistently activated, causing a chronic state of cellular stress, such as the chronic inflammation. This persistent inflammation state, or non-resolved inflammation, can contribute directly or indirectly to the etiology of the most common of neurodegenerative diseases, including PD. In fact, immune cells are in general more reactive during aging, in a state called “primed,” being more susceptive to secondary inflammatory stimulus. This is the case of aged-microglia, which behavior is overexcitable and resistant to regulation, causing an amplified immune reaction in the CNS (189, 190). Also, the activation of microglia is influenced by astrocytes and neurons, in a cell-to-cell interaction, direct or indirectly through cytokines and neurotransmitters. The initial activation of the innate immune system may have protective roles, but when these innate defense mechanisms become dysregulated and maladaptive, it leads to disease progression. A possible scenario could be that chronic circulating inflammatory cytokines derived from glial cells, from blood-derived immune cells and/or from an imbalanced microbiota in the progress of aging can result in a non-autonomous degeneration of dopaminergic neurons in PD. For instance, necroptosis, a different mechanism of cell death, is triggered by an excessive inflammatory response, especially due to TNF-α signaling. In a recent study, it was demonstrated that the dopaminergic cell death induced by treatment with 6-OHDA *in vitro* (191) or MPTP *in vivo* (192) was blocked by the pre-treatment with an inhibitor of necroptosis (necrostatin-1). Moreover, a recently published paper links the lack of *PINK1* in glial cells with enhanced inflammation-induced neuronal death in an *in vivo* model of PD (39). The understanding of the contribution of these cells in the etiology and/or progression of PD will support the design of more effective lines of treatment for this devasting pathology.

## Author contributions

PT-E, AP, and MN drafted the contents of this review, and together with RV wrote the text. All authors contributed equally to the critical reading of the final manuscript, including text and figures.

### Conflict of interest statement

The authors declare that the research was conducted in the absence of any commercial or financial relationships that could be construed as a potential conflict of interest.
